# A novel method for quantifying the biomechanical parameters of orbital soft tissue using a corneal dynamic scheimpflug analyser: a retrospective study

**DOI:** 10.1186/s12886-019-1064-7

**Published:** 2019-02-18

**Authors:** Ho Sik Hwang, Eun Chul Kim, Man Soo Kim, Suk-Woo Yang

**Affiliations:** 1 Department of Ophthalmology, Chuncheon Sacred Heart Hospital, Hallym University, Chuncheon, South Korea; 2 Department of Ophthalmology, Bucheon St. Mary’s Hospital, Catholic University of Korea, Bucheon, South Korea; 3 Department of Ophthalmology, Seoul St. Mary’s Hospital, Catholic University of Korea, Banpodaero 222, Seocho-gu, Seoul, South Korea

**Keywords:** Corneal dynamic scheimpflug analyser, Corvis ST, Graves ophthalmopathy, Orbital soft tissue, Whole eye movement

## Abstract

**Background:**

To demonstrate that the Corvis ST could be used to quantify the biomechanical parameters of the orbital soft tissues by measuring and comparing whole eye movement (WEM) using the Corvis in normal eyes and in eyes of patients with Graves ophthalmopathy.

**Methods:**

Forty four eyes of 44 ophthalmologically normal subjects and 28 eyes of 28 patients with Graves ophthalmopathy were included in the study. After Corvis test, the examiners recorded WEM by air puff. In the patients with Graves ophthalmopathy, the partial correlation coefficient adjusted for age and gender was calculated to analyze the correlation between exopthalmometry and WEM. Same analysis was repeated for the correlation between and the cross sectional area (%) of the extraocular muscles in the orbit CT and WEM.

**Results:**

WEM was 0.314 ± 0.083 mm in the normal subjects and 0.227 ± 0.079 mm in the Graves ophthalmopathy group (*p* = 0.000). The exophthalmometry was not significantly correlated with WEM after adjusting for age and gender (R = 0.083, *p* = 0.688). In the 21 Graves ophthalmopathy patients examined by orbit CT, after adjusting for age and gender, WEM significantly decreased as the cross sectional area (%) of the extraocular muscles in the orbit increased (R = − 0.461, *p* = 0.047).

**Conclusions:**

WEM by Corvis could be used to quantify the biomechanical parameters of the orbital soft tissue. However, it is unclear whether WEM effectively represents the orbital biomechanical parameters, because WEM is only 0.6% of the orbital depth.

**Electronic supplementary material:**

The online version of this article (10.1186/s12886-019-1064-7) contains supplementary material, which is available to authorized users.

## Background

The recently developed corneal dynamic scheimpflug analyser (Corvis ST; Oculus, Wetzler, Germany) is a diagnostic instrument comprising a noncontact tonometer, a Scheimpflug geometry, and an ultra-high speed camera that can measure intraocular pressure and corneal biomechanical parameters. The Corvis ST releases a puff of air onto the patient’s cornea just like a noncontact tonometer. The Scheimpflug geometry and ultra high-speed camera record a movie of the change in the corneal sagittal section in response to the air puff. When the air puff is released, the cornea first flattens, then becomes somewhat concave, flattens again, and then returns to its original contour. The Corvis ST measures not only intraocular pressure, but several other parameters as well, such as the deformation amplitude and first applanation time, which represent corneal biomechanical parameters. This device is therefore used in clinics and for studies of corneal biomechanics [1–6], keratoconus [7–10], changes after refractive surgery [11–15], and glaucoma [16–20].

During the measurement, there is a slight but significant movement of the whole eye globe [21]. As the cornea deforms and approaches maximum displacement, the whole eye displays a slow linear motion in the anterior-posterior direction. When the cornea reaches maximum displacement, the whole eye motion becomes more pronounced and nonlinear in nature, as the air puff pressure continues to increase to a consistent maximum.

The authors hypothesized that we could use the Corvis ST to quantify the biomechanical parameters of the orbital soft tissue behind the eyeball based on the eyeball displacement during the air puff. If the orbital soft tissues, such as the fat and extraocular muscles, change, we would expect that eyeball displacement would also change.

In the present study, the authors measured and compared whole eye movement (WEM) using the Corvis ST in normal eyes and in eyes of patients with Graves ophthalmopathy whose orbital soft tissue might be altered. The authors demonstrated that the Corvis ST could be used to quantify the biomechanical parameters of the orbital soft tissues.

## Methods

Corvis ST data collected during intraocular pressure measurement of patients who visited oculoplastics clinic of Seoul St. Mary’s Hospital were used in this study. A total of 44 right eyes of 44 ophthalmologically normal subjects and 28 right eyes of 28 patients with Graves ophthalmopathy were included in the study. This retrospective study adhered to the tenets of the Declaration of Helsinki and received institutional review board approval from Seoul St. Mary’s Hospital for the analysis of their medical records. This study was conducted from January 2016 to December 2016. The authors collected age, gender and Corvis ST data of normal subjects and patients with Graves ophthalmopathy and exophthalmometry, severity and activity of Graves ophthalmopathy and orbit computed tomography (CT) scans of patients with Graves ophthalmopathy. One author had access to information that could identify individual participants during or after data collection. Inclusion criteria for the normal group included the absence of the following: thyroid disease, orbital inflammation, blow out fracture, severe lid inflammation, corneal ulcer, and risk of eyeball rupture (severe thinning of cornea or sclera). The inclusion criterion for the Graves ophthalmopathy group was mild severity or higher according to European Group on Graves’ orbitopathy (EUGOGO) [22]. Patients who had received radiation therapy or systemic steroid treatment were included. Patients with a risk of eyeball rupture or who had undergone decompression surgery for Graves ophthalmopathy were excluded.

The examiners performed the Corvis ST test for intraocular pressure measurement. The examiners examined the eyes with a biomicroscope before the Corvis ST test, and confirmed the absence of exclusion criteria, such as the risk of eyeball rupture. The examiners performed the Corvis ST test as follows: the eye was positioned in front of the system at a distance of 11 mm between the corneal apex and the air tube. When the eye was aligned and the Scheimpflug image was in focus, the air puff was automatically released and the cornea was imaged during the deformation event.

**Fig. 1:**
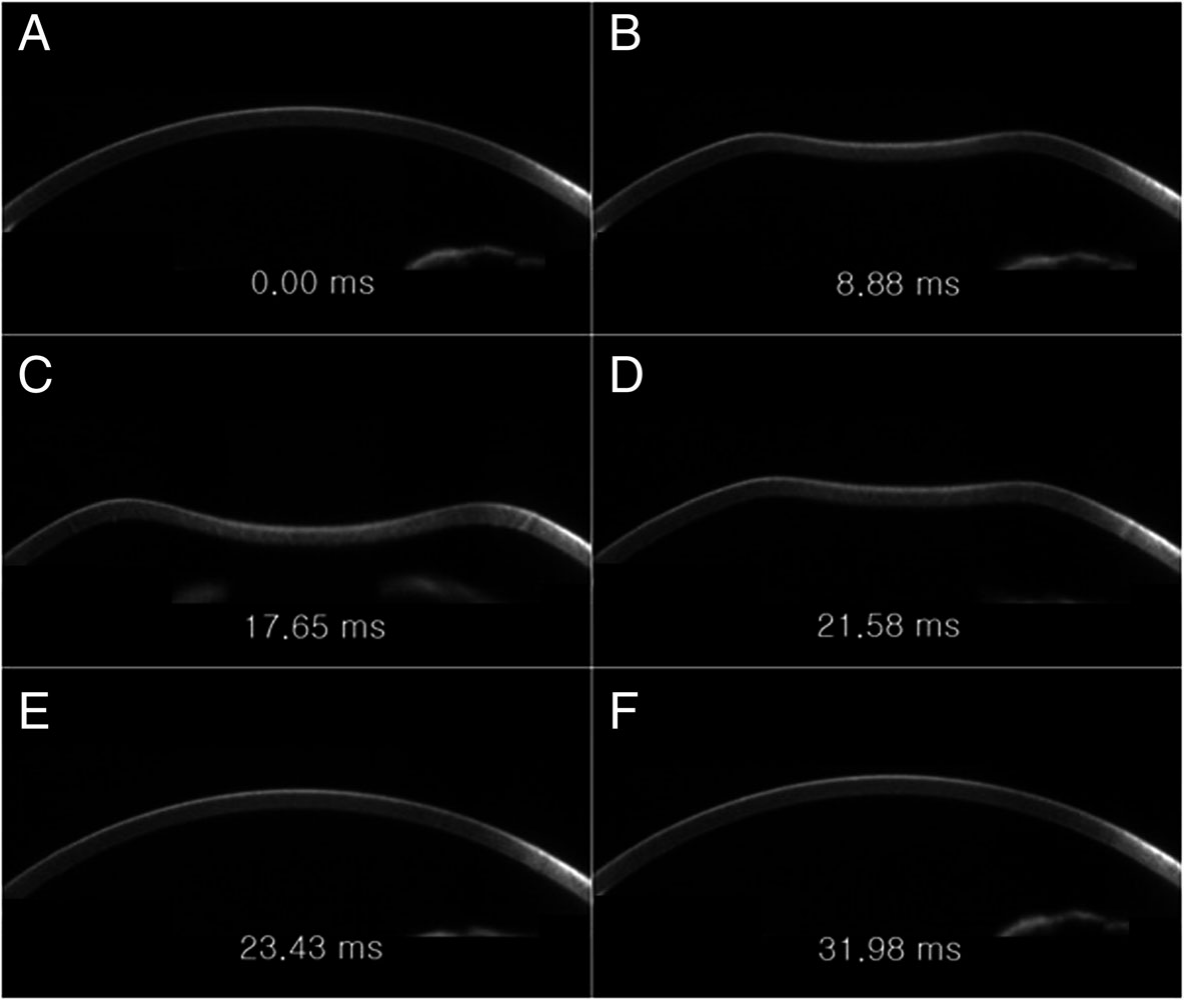
Whole eye movement during Corvis test. The movies showed that when the air puff was released (**a**), the cornea became concave (**b**) and the eyeball moved backward a little. After maximal deformation of the cornea (**c**), the corneal deformation decreased (D), and when the cornea returned to its original contour (**e**), the eyeball moved forward again (**f**)


The authors recorded the central corneal thickness measured by the Corvis ST. The authors found that upon release of the air puff, the eyeball itself moves backward a little, and after the cornea returns to its original contour, the eyeball moves forward again (Fig. 1, Additional file 1: Video S1). The new analysis software (version 1.5r1902) was used to measure whole eye movement [21, 23].

**Additional file 1: Movie S1.** Eyeball displacement during an air puff. This movie showed that when the air puff was released, the cornea became concave and the eyeball moved backward a little. After maximal deformation of the cornea, the corneal deformation decreased, and when the cornea returned to its original contour, the eyeball moved forward again. (WMV 799 kb)


In the patients with Graves ophthalmopathy, the examiners performed exophthalmometry using a Hertel Exophthalmometer (K-0161, Inami, Fukuoka, Japan) and an author classified Graves’s ophthalmopathy severity according to EUGOGO [22] and assessed activity according to Clinical Activity Score (CAS) [24] for each severity subgroup. One experienced oculoplastics specialist (YSW) classified the patients. The cross-sectional area of extraocular muscle in the orbit by CT was calculated. Among the 28 patients with Graves’s ophthalmopathy, 21 underwent orbit CT scans. A coronal slice 6 mm behind the posterior pole was selected and the cross-sectional area of the orbit and four rectus muscles were measured. Next, the cross sectional area (%) of the four rectus muscles in the orbit was calculated according to the study by Kim et al. [25].

### Statistical analyses

The mean and standard deviation of WEM in the normal group were calculated. In the normal group, a Kolmogorov-Smirnov one-sample test was applied to evaluate the normality of the distribution of WEM measurements. A non-parametric Spearman correlation test was used to examine the relation between age and WEM in the normal group. A t-test was used to compare WEM between male and female in the normal group. Age (Mann-Whitney U-test), gender (Fisher’s exact test), central corneal thickness (Mann-Whitney U-test), and WEM (Mann-Whitney U-test) between the normal group and patients with Graves ophthalmopathy were compared. In the patients with Graves ophthalmopathy, the partial correlation coefficient adjusted for age and gender was calculated to analyze the correlation between exopthalmometry and WEM. Same analysis was repeated for the correlation between and the cross sectional area (%) of the extraocular muscles in the orbit CT and WEM. The mean WEM values were compared among mild, moderate to severe and sight-threatening groups by analysis of variance (ANOVA). The authors used SPSS 18.0 for statistical analysis and considered *p* < 0.05 to indicate statistical significance.

## Results

### Patient characteristics

Table 1 shows the demographics and characteristics of the 44 eyes from the 44 normal subjects and the 28 eyes of the 28 patients with Graves ophthalmopathy.

**Table 1:** Demographics and characteristics of normal and Grave’s ophthalmopathy group

	Normal group	Grave’s ophthalmopathy group	*p*-value
Number of subjects	44	28	
Number of eyes	44	28	
Age (years)	54.8 ± 17.7	51.2 ± 16.2	0.225 (u-test)
Gender (Male: Female)	14:30	7:21	0.774 (Fisher’s exact test)
Central corneal thickness (μm)	546 ± 36	551 ± 41	0.317 (u-test)
WEM (mm)	0.314 ± 0.083	0.227 ± 0.079	0.000 (u-test)

### Whole eye movement

**Fig. 2:**
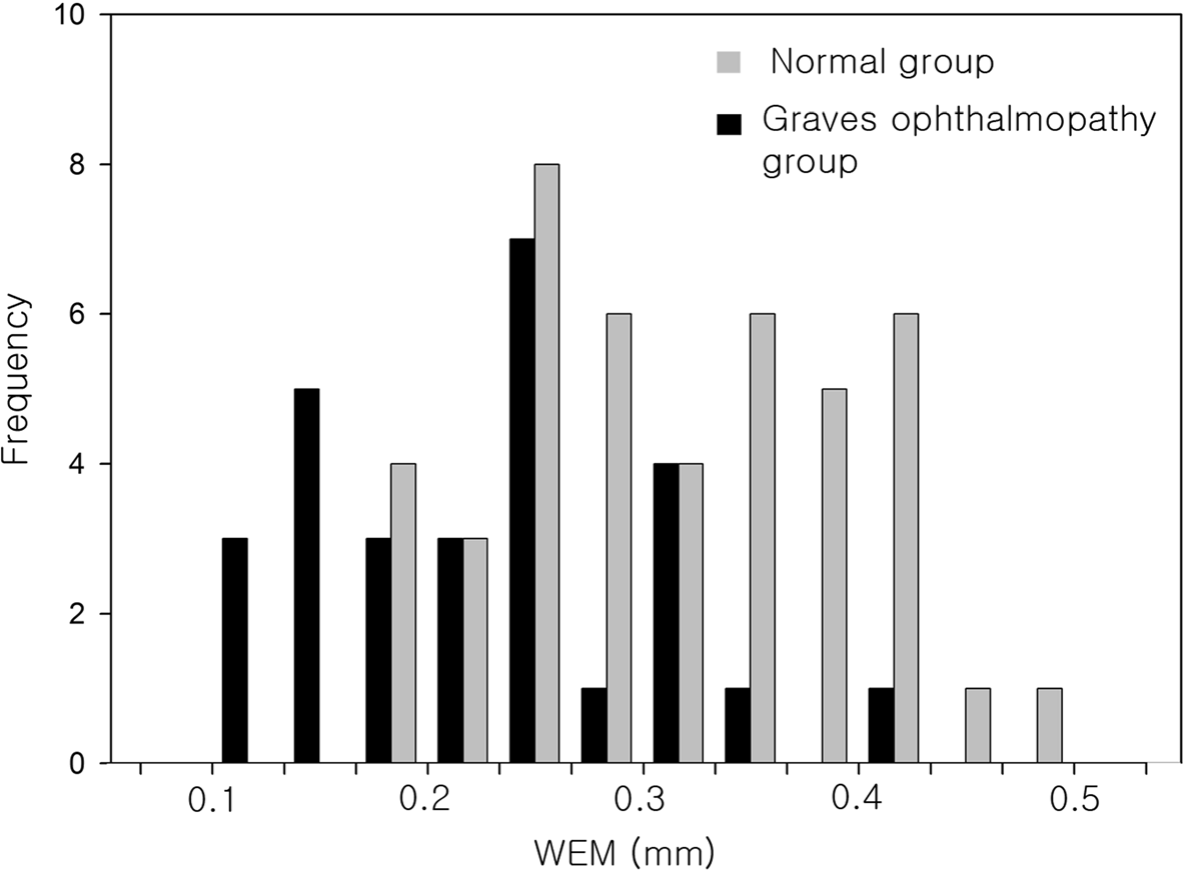
The distribution of whole eye movement (WEM) in the normal subjects and Graves ophthalmopathy group. The mean WEM was 0.314 ± 0.083 mm (range, 0.174–0.492 mm) in the normal subjects and the values were fairly normally distributed (*P =* 0.200) based on the Kolmogorov-Smirnov one-sample test. In the Graves ophthalmopathy group, the mean WEM was 0.227 ± 0.079 mm (range, 0.110–0.429 mm). WEM was significantly smaller in the Graves ophthalmopathy group compared with the normal group (Mann Whitney U-test, *p* = 0.000)

Figure 2 shows the distribution of WEM in the normal subjects and Graves ophthalmopathy group. The mean WEM was 0.314 ± 0.083 mm (range, 0.174–0.492 mm) in the normal subjects and the values were fairly normally distributed (*P =* 0.200; Fig. 2) based on the Kolmogorov-Smirnov one-sample test. In the Graves ophthalmopathy group, the mean WEM was 0.227 ± 0.079 mm (range, 0.110–0.429 mm). WEM was significantly smaller in the Graves ophthalmopathy group compared with the normal group (Mann Whitney U-test, *p* = 0.000).

### Relationship with age and gender

**Fig. 3:**
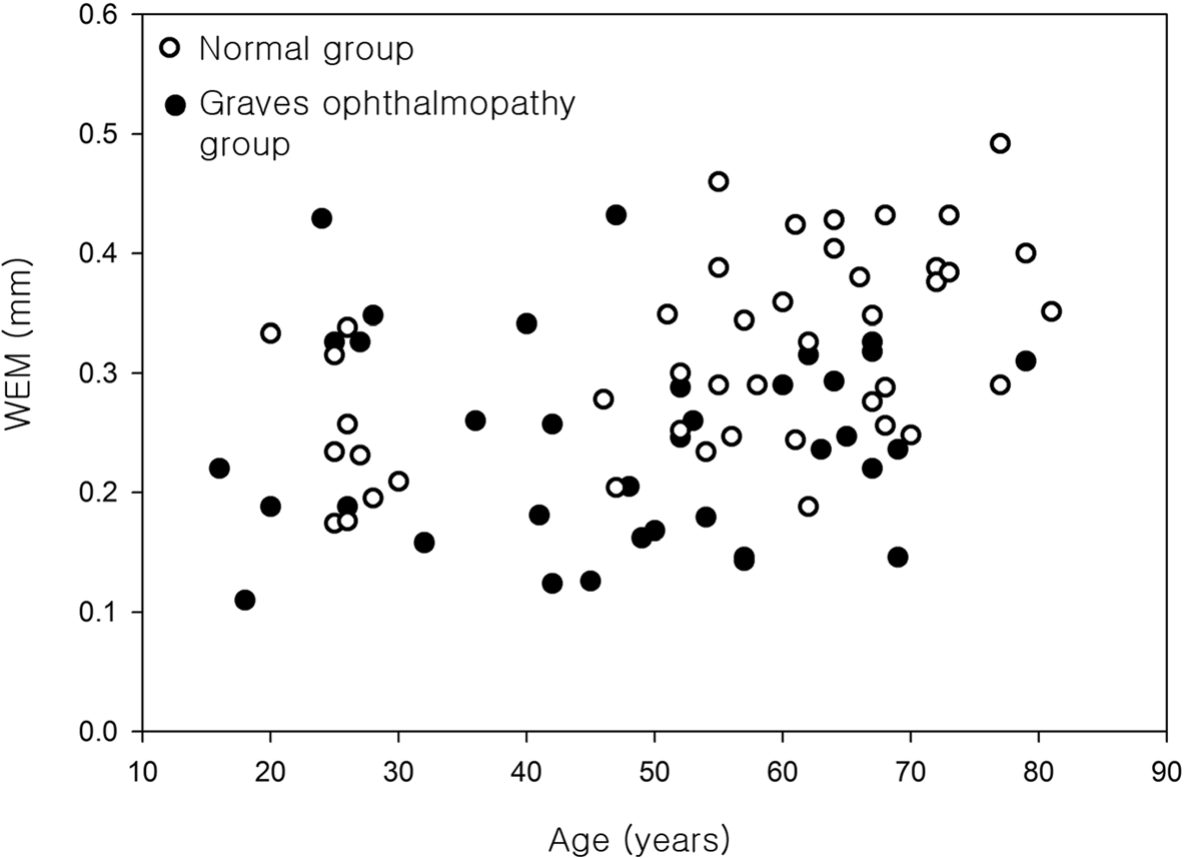
The correlation between eyeball displacement and age in the normal group and Graves ophthalmopathy group. In the normal group, whole eye movement was significantly positively correlated with age (Spearman correlation coefficient *r* = 0.531, *p* = 0.000)

**Fig. 4:**
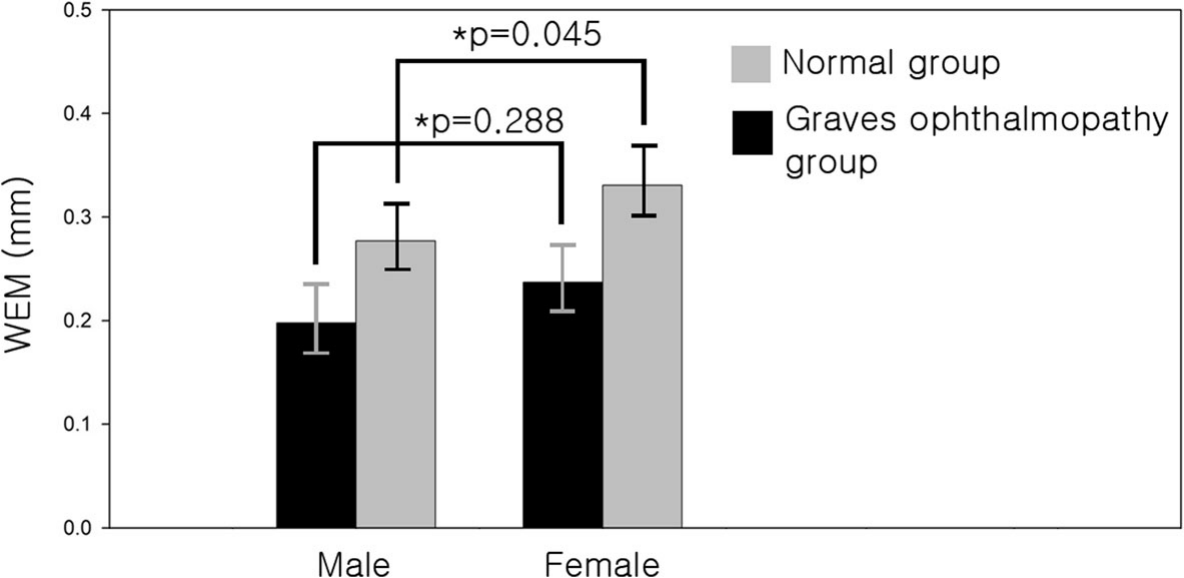
Comparison of mean eyeball displacement between male and female in healthy subjects and Graves ophthalmopathy group. Whole eye movement (WEM) in the male was 0.277 ± 0.076 mm and that in the female was 0.331 ± 0.081 mm (u-test, *p* = 0.045). In the Graves ophthalmopathy group, WEM was 0.198 ± 0.081 mm in the male and 0.237 ± 0.077 mm in the female (u-test, *p* = 0.288)

In the normal group, WEM was significantly positively correlated with age (Spearman correlation coefficient r = 0.531, p = 0.000, Fig. 3). Mean age of the 14 normal male subjects was 49.2 ± 20.0 years and mean age of 30 normal female subjects was 57.3 ± 16.5 years, with no significant difference between them (u-test *p* = 0.262). WEM in the male was 0.277 ± 0.076 mm and that in the female was 0.331 ± 0.081 mm (u-test, *p* = 0.045) (Fig. 4). In the Graves ophthalmopathy group, WEM was 0.198 ± 0.081 mm in the male and 0.237 ± 0.077 mm in the female (u-test, *p* = 0.288).

### Graves ophthalmopathy

**Fig. 5:**
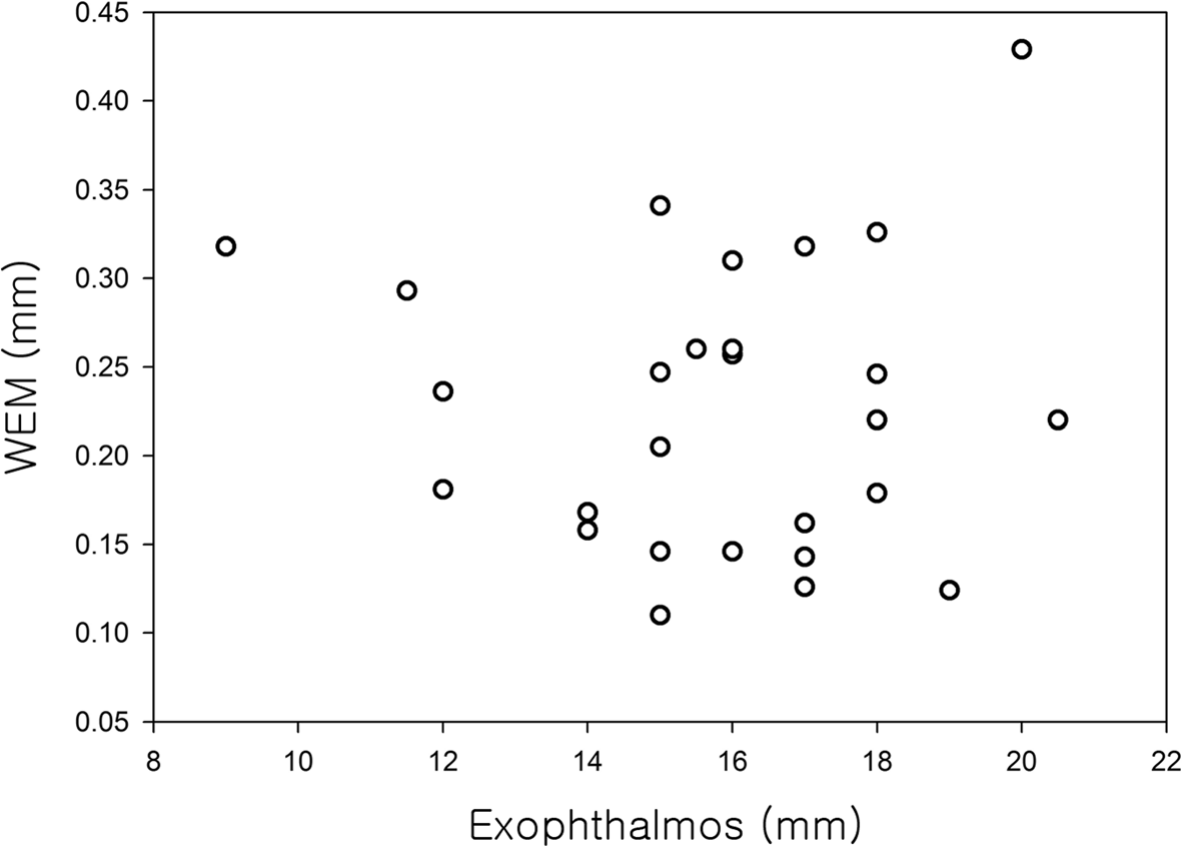
The correlation between whole eye movement (WEM) and exopthalmometry in the 28 eyes of the 28 Graves ophthalmopathy patients. WEM tended to decrease as exophthalmometry increased, but the correlation was not statistically significant after adjusting for age and gender (R = 0.083, *p* = 0.688)

**Fig. 6:**
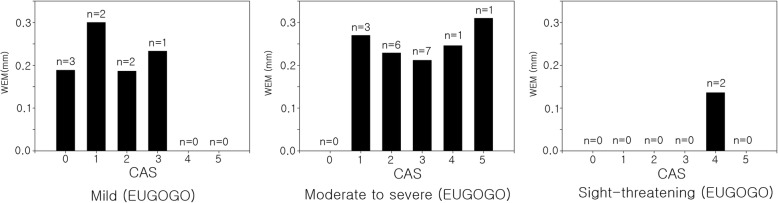
Whole eye movement **(**WEM) versus clinical activity score (CAS) in each severity subgroups. The mean WEM was 0.233 ± 0.077 mm in mild severity group, 0.235 ± 0.080 mm in moderate to severe group and 0.227 ± 0.079 mm in sight –threatening group. There was no significant differences between the groups (ANOVA, *p* = 0.244)

Figure 5 shows the correlation between WEM and exopthalmometry in the 28 eyes of the 28 Graves ophthalmopathy patients. WEM tended to decrease as exophthalmometry increased, but the correlation was not statistically significant after adjusting for age and gender (R = 0.083, *p* = 0.688); Fig. 5). The mean WEM was 0.233 ± 0.077 mm in mild severity group, 0.235 ± 0.080 mm in moderate to severe group and 0.227 ± 0.079 mm in sight –threatening group. There was no significant differences among the groups (ANOVA, *p* = 0.244). Figure 6 shows mean WEM versus CAS in each severity subgroups.

**Fig. 7:**
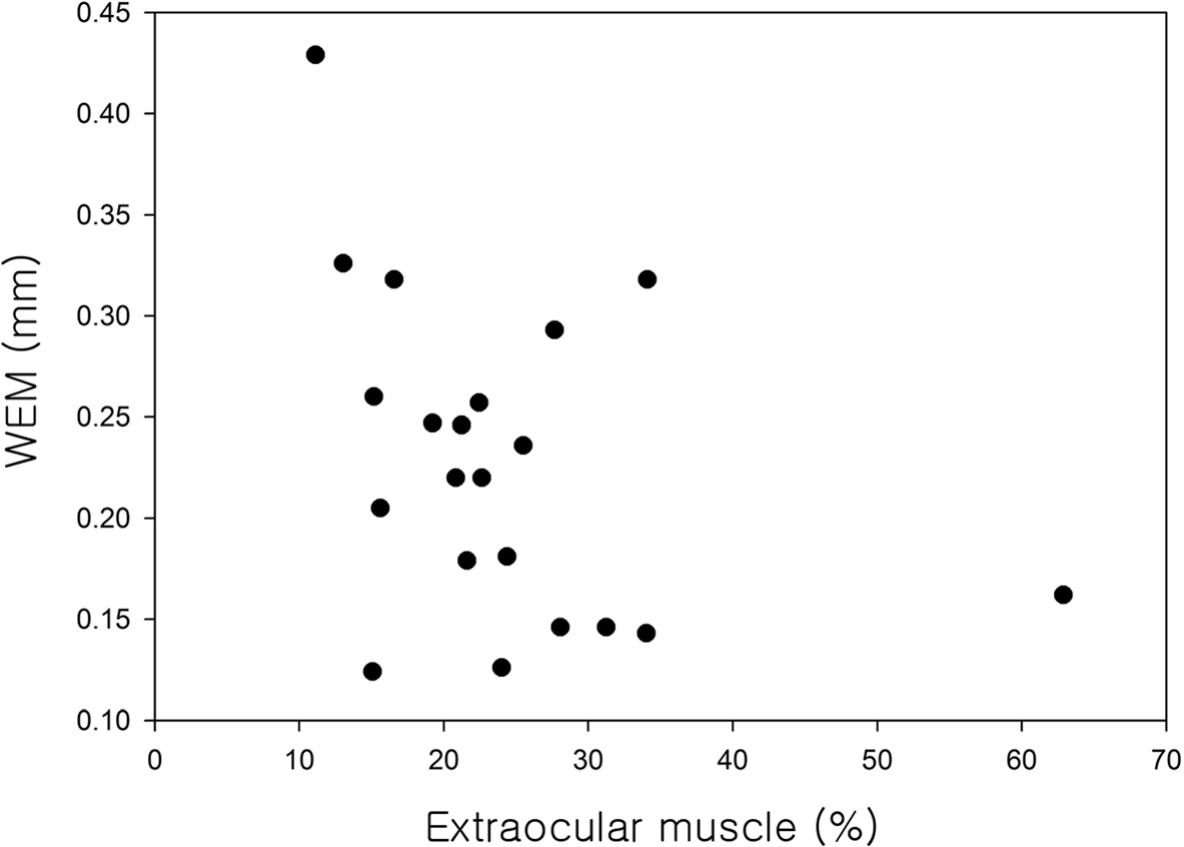
The correlation between whole eye movement (WEM) and the cross sectional area (%) of the extraocular muscles in the 21 Graves ophthalmopathy patients examined by orbit computed tomography (CT). After adjusting for age and gender, WEM significantly decreased as the cross sectional area (%) of the extraocular muscles in the orbit increased (R = − 0.464, p = 0.045)

In the 21 Graves ophthalmopathy patients examined by orbit CT, after adjusting for age and gender, WEM significantly decreased as the cross sectional area (%) of the extraocular muscles in the orbit increased (R = − 0.464, *p* = 0.045; Fig. 7).

## Discussions

The Corvis ST is a diagnostic instrument originally developed to measure biomechanical response to an air puff by analyzing the shape of the deformation in the images, as well as the timing of specific events. By viewing the ultra-high speed video by the Corvis ST, the authors observed WEM induced by the air puff release and therefore evaluated whether the Corvis ST could be also used to assess the biomechanical parameters of the orbital soft tissue. WEM induced by the air puff increased with increasing age and was greater in female than in male. WEM was smaller in patients with Graves ophthalmopathy than in normal subjects.

Recently clinical research papers on WEM have been published. Vinciguerra et al. measured WEM in healthy subjects and obtained normative data. They found that WEM increases with age [21]. This is consistent with the results of the present study. WEM was used to develop a new index for keratoconus detection [23, 26, 27]. WEM was also used in glaucoma research. Jung et al. found that unadjusted WEM was smaller in glaucoma group compared to normal group [28]. Aoki et al. measured WEM in open angle glaucoma and obtained the relationship between WEM and axial length of eyeball [29].

In the present study, WEM in patients with Graves ophthalmopathy was smaller than that in normal subjects. In other words, orbital soft tissue is stiffer and less compliant in Graves ophthalmopathy patients than in normal subjects. It means a retrobulbar resistance as a clinical sign in Graves ophthalmopathy. A previous study reported that orbital compliance is decreased in Graves ophthalmopathy patients [30]. In that research, greater force was required to push the eyeball the same distance in Graves ophthalmopathy patients as in normal controls. The mean forward force for 3 mm retropulsion of the eye was 70.1 g in normal subjects, 102.5 g in compressive optic neuropathy, and 56.7 g in stable Graves ophthalmopathy. In Graves ophthalmopathy patients, the orbital fat becomes fibrotic [31] and the extraocular muscle accumulates mucopolysaccharides and become larger. These factors are thought to increase the stiffness of orbital soft tissue.

Recently, Vellara et al. [32] used the Corvis ST to assess the orbital compliance in patients with thyroid eye disease (TED) and compared the results with a cohort of healthy subjects. They investigated the feasibility of using measurements derived from the Corvis ST as a diagnostic tool for TED. They used Matlab (Natick, VA, V.8.4.0.150421 (R2014b)) for measurement of maximum orbital deformation (MOD). They measured MOD of 20 eyes of 20 patients with TED and 152 eyes of 152 healthy subjects using Corvis ST. The mean MOD was 0.16 ± 0.04 mm and 0.25 ± 0.05 mm for TED and healthy eyes, respectively (*p* < 0.001). The MOD values are smaller than WEM in the present study. The authors think that the difference of ethnicity between two studies might cause the different results. Eyes included in the study (20 eyes with TED and 152 healthy eyes) were incorporated into a receiver operating characteristic (ROC) analysis. A cut-off value of 0.18 mm yielded 92% sensitivity and 84% specificity.

Their research and ours have something in common. Eyeball displacement using Corvis ST was compared between patients with Graves ophthalmopathy and healthy subjects. These eyeball displacement was interpreted as an orbital compliance. The authors have some advances over their research. First, the authors analyzed correlation between WEM and clinical parameters such as exophalmometry, disease severity and activity, and the cross sectional area (%) of the extraocular muscles in the orbit. Second, the authors measured WEM using Corvis software instead of Matlab, so clinical ophthalmologists can easily use this method; Third, there is no significant difference of age and ethnicity between two groups unlike their research. Fourth, the authors obtained distribution of WEM (histrogram) in healthy subjects.

Leszczynska et al. investigated orbital biomechanical properties in patients with thyroid orbitopathy and in age and gender-matched healthy subjects using the Corvis-ST. [33] Whole eye movement length (WEMl) were measured in 39 patients with thyroid orbitopathy (= group I) and in 33 age- and gender-matched healthy subjects (= group II) using the CST. There was a statistically significant difference between both groups in mean WEMl (207 ± 57 vs. 322 ± 50 μm) (*p* = 0.01). WEMl values of each group were similar to our results and the mean WEMI of thyroid orbitopathy group was smaller than that of healthy group like the present study.

Eyeball displacement measured using the Corvis ST is true eyeball movement and not just axial shortening and elongation of eyeball induced by the air puff based on the fact that the eyeballs moved forward again after returning to their original corneal contour (S1Video). This finding is consistent with the findings of previous studies [21, 23, 26–29, 33, 34].

In the normal subject group, mean WEM was 0.314 ± 0.083 mm, which was 57.5% of mean central corneal thickness (546 μm). Mean orbital depth is 45–55 mm [35], so mean eyeball displacement was just 0.6% of the orbital depth. In normal adults, the mean volume of the orbit is 29.7 ml and the volume of the eyeball is 6.5 ml. Therefore, the volume of the orbit that does not include the eyeball is 23.2 ml [35]. Fat is the most predominant tissue in the orbit. Most of the rest of the orbit comprises extraocular muscle. Therefore, WEM measured by the Corvis ST represents the biomechanical parameters of 23.2 ml of fat and extraocular muscle in the orbit.

Eyeball displacement appears to decrease as exophthalmos increases, but after adjusting for age and gender, the authors found no significant correlation (R = 0.083, *p* = 0.688). WEM showed no significant differences among mild, moderate to severe and sight-threatening groups (ANOVA, *p* = 0.244). In 21 patients with Graves ophthalmopathy examined by orbital CT, WEM decreased as the cross sectional area (%) of the extraocular muscles in the orbit increased, after adjusting for age and gender (R = − 0.461, *p* = 0.047). That is, as the cross sectional area (%) of the extraocular muscles in the orbit increases, the orbit becomes stiffer and eyeball displacement decreases.

How can the biomechanical parameters of orbital soft tissue measured using the Corvis ST be applied clinically? The factors that contribute to WEM may be the state of the orbital fat and extraocular muscles, and the presence or absence of blow out fracture. In the present study, WEM decreased as the cross sectional area (%) of the extraocular muscles increased in patients with Graves ophthalmopathy. These findings indicate that WEM can be used as a measure to monitor orbital soft tissue stiffness resulting from Graves ophthalmopathy. Changes in the extraocular muscle can be examined by CT. CT scanning cannot be performed frequently, however, due to both the risk of radiation and the high cost. The Corvis ST is a simple and easy-to-perform method for evaluating orbital soft tissue and the progression of Graves ophthalmopathy during follow-up.

Advantages of using the Corvis ST to quantify the biomechanical parameters of the orbital soft tissue include its easy application and lack of discomfort for the subjects. And a recent study demonstrated good repeatability [36]. But, it is unclear whether eyeball displacement effectively represents the orbital biomechanical parameters because WEM is only 0.6% of the orbital depth.

## Conclusions

In conclusion, the authors used the Corvis ST, which was developed to measure the biomechanical parameters of the cornea, to quantify the biomechanical parameters of the orbital soft tissue.

## Abbreviations

ANOVA: Analysis of variance
CAS: Clinical Activity Score
CT: Computer tomography
EUGOGO: European Group on Graves’ orbitopathy
MOD: Maximum orbital deformation
ROC: Receiver operating characteristic
TED: Thyroid eye disease
WEM: Whole eye movement
WEMl: Whole eye movement length
